# Associations between knee extensor strength and gait characteristics derived from shoe-based wearable sensors in older women

**DOI:** 10.1038/s41598-026-51573-0

**Published:** 2026-05-03

**Authors:** Takuma Inai, Shoma Kudo, Wakako Tsuchida, Masahiro Fujimoto

**Affiliations:** 1 Integrated Research Center for Self-Care Technology, National Institute of Advanced Industrial Science and Technology (AIST), Takamatsu, Kagawa Japan; 2 Health and Medical Research Institute, National Institute of Advanced Industrial Science and Technology (AIST), Takamatsu, Kagawa Japan; 3 Research Institute on Human and Societal Augmentation, National Institute of Advanced Industrial Science and Technology (AIST), Kashiwa, Chiba Japan; 4 Integrated Research Center for Self-Care Technology, National Institute of Advanced Industrial Science and Technology (AIST), 2217-14 Hayashi-cho, Takamatsu, 761-0395 Kagawa Japan

**Keywords:** Walking, Isometric knee extensor strength, Insole force sensor, Inertial measurement unit, Ground reaction force, Health care, Medical research, Rheumatology

## Abstract

Declining knee extensor strength (KES) is a recognized risk factor for musculoskeletal disorders including knee osteoarthritis. Regular self-assessment of knee extensor strength in older women is essential for prevention of the progression. This study aimed to clarify the relationship between the KES and parameters derived from shoe-based wearable sensors during natural walking. Thirty-seven older women participated in this study. Insole force sensors and shoe-mounted IMU sensors were used to obtain spatiotemporal, ground reaction force (GRF), and foot pitch angle parameters. The maximal isometric KES was measured, and simple and partial correlations (controlling for gait speed and age) were calculated. KES was significantly correlated with (1) percentages of the stance and double-stance phases (*r* = -0.58 to -0.46), (2) timing of the first peak of the vertical GRF (*r* = -0.57), and (3) foot pitch angle at toe-off (*r* = -0.56). Furthermore, these parameters remained significantly associated with the KES after controlling for gait speed and age. These findings may contribute to the development of wearable sensor–based systems to estimate and quantify knee extensor strength using daily walking data.

**Supplementary Information:**

The online version contains supplementary material available at 10.1038/s41598-026-51573-0.

## Introduction

Regular assessment of knee extensor strength (KES) is essential, because a decline in strength is a known risk factor for musculoskeletal disorders. Previous studies^1–3^ have reported that knee extensor muscle weakness is associated with an increased risk of developing knee osteoarthritis, particularly among older women^4^. Therefore, regular self-assessment of the KES in older women is critical for preventing the onset of musculoskeletal disorders.

Devices such as the Biodex system^5^ and handheld dynamometers^6^ are commonly used in research and clinical settings to evaluate the KES. Although the Biodex system^5^ is widely used in research owing to its high reproducibility, it is expensive. In contrast, handheld dynamometers are inexpensive and portable, making them suitable for use in research and healthcare facilities^7^. However, older women who have not yet developed musculoskeletal disorders such as knee osteoarthritis are unlikely to visit healthcare facilities regularly, underscoring the need for voluntary and regular assessments of KES at home or through community health programs. Furthermore, maintaining regular self-assessment can be challenging for older adults with diminished health motivation. Clarifying the relationship between KES and gait may help develop a system that can naturally and indirectly estimate the KES based on daily walking activities. This combined approach could help monitor KES in older women without knee osteoarthritis, who are unlikely to undergo medical screening, potentially contributing to the early detection of declines in KES and onset of knee osteoarthritis.

Recent advances in wearable sensors (e.g., insole force sensors^8^ and inertial measurement units [IMUs]^9–11)^ have enabled the collection of gait-related data during daily walking by attaching sensors to shoes. Insole force sensors such as Loadsol^®^ can measure vertical ground reaction forces (GRFs)^8^, therefore, we can calculate temporal and GRF parameters (e.g., the first peak). IMU sensors attached to shoes can measure acceleration and angular velocity, and calculate spatiotemporal and kinematic parameters (e.g., foot pitch angle)^9–11^. Previous studies^12–27^ have measured KES and gait, and several relationships between KES and parameters have been identified (e.g., gait speed^13,14,16,25^, stride length^14,27^, and the first peak of GRF^12)^. However, these studies used a motion capture system^14,19,27^, an instrumented treadmill^12,14^ and walkway^13,16,18,20^, a photocell system^15^, electronic phototubes^21,26^, or a stopwatch^14,17,22–25^(although not explicitly stated, it is assumed that the previous studies^14,22,24,25^ used a stopwatch [or electronic phototubes] to measure gait speed). None of the previous studies used shoe-based sensors.

To our knowledge, no study has examined simple correlations between the KES and spatiotemporal, ground reaction force, or foot pitch angle parameters, derived from shoe-based wearable sensors, in older women. Additionally, although gait speed^28,29^ may affect various parameters (amplitudes of spatiotemporal gait parameters, joint kinematics, joint kinetics, and GRFs), the relationships between the KES and sensor-derived parameters after controlling for gait speed (i.e., partial correlations) remain unclear. Clarifying these associations may be useful for estimating the KES from the parameters obtained using wearable sensors.

Therefore, this study aimed to explore the associations (simple and partial correlations) between KES and spatiotemporal, GRF, and foot pitch angle parameters derived from wearable sensors in elderly women. Previously, KES has been reported to be associated with gait speed^13,14,16,25^, and gait speed has been shown to be associated with e.g. step length^30^(approximately half of the stride length) and the first peak of the vertical GRF^12^. Therefore, we hypothesized that the parameters derived from shoe-based wearable sensors were associated with KES (first hypothesis). Moreover, we hypothesized that these associations would not remain significant in the partial correlation analyses after controlling for gait speed as a confounding factor (second hypothesis).

## Methods

### Participants

The sample size for the correlation analyses was determined using R language 4.3.0 (R Core Team, Vienna, Austria). An a priori power analysis was conducted with the following parameters: effect size (Cohen’s r) = 0.462, significance level (α) = 0.05, and statistical power (1 − β) = 0.80. This effect size was based on a previous study^25^ that reported a correlation of 0.462 between the maximal isometric KES and gait speed in older women. The required sample size was 34.

Thirty-seven older women participated in this study (age: 78.1 ± 4.1 years, height: 1.51 ± 0.06 m, body mass: 50.1 ± 9.5 kg, body mass index: 22.4 ± 3.5). The inclusion criteria were as follows: (1) age > 65 years and (2) ability to walk 20 m without a walking aid. Participants were excluded if they (1) regularly engaged in professional competitive sports, (2) had visual impairments, (3) had severe hearing impairments that prevented oral communication, (4) had undergone a bone fracture or surgery within the past year, (5) had neurological disorders affecting motor or sensory function or vestibular dysfunction, (6) took sedatives such as anticonvulsants or anti-anxiety medications daily, or (7) had implanted medical electrical devices such as a cardiac pacemaker. According to previous studies, visual impairment^31^, a history of fracture and surgical history (e.g., ankle fracture and surgery^32^), neurological disorders^33^, vestibular dysfunction^34^, and the use of sedatives^35,36^ may affect gait. Therefore, we established these conditions as exclusion criteria. Individuals who regularly engage in professional sports activities may have high muscle strength (i.e., potential outliers); therefore, those who regularly participated in professional sports were excluded from the analysis. Considering the potential cardiac load during the assessment of maximal KES, participants with implanted cardiac pacemakers were excluded.

The study adhered to the principles of the Declaration of Helsinki. The study protocol was approved by the Ethics Committee of National Institute of Advanced Industrial Science and Technology (IRB number: hi2023-0532). All participants provided written informed consent before participation.

## Experiments

### Gait

The participants were instructed to walk back and forth along a 15-m straight pathway five times at a self-selected comfortable pace. Before these trials, the participants completed several practice walks along the pathway to familiarize themselves with the tasks.

**Fig. 1:**
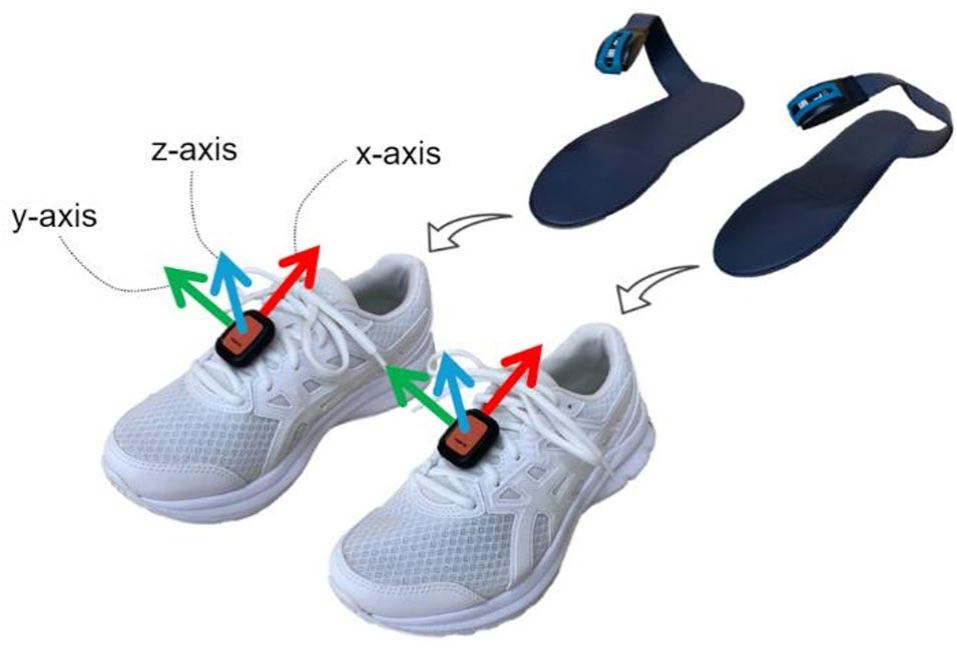
Insole force sensors and IMUs. Insole force sensors were inserted into both shoes. The IMUs were attached to the dorsum of both shoes; the x-axis is oriented posteriorly, y-axis is oriented to the right, and z-axis is oriented superiorly.

Shoes (JOLT3, ASICS Corp., Hyogo, Japan) were prepared in sizes ranging from 22.0 cm to 28.0 cm, in 0.5 cm increments. Each participant selected the pair that provided a comfortable fit. Insole force sensors (Loadsol Pro; Novel GmbH Inc., Munich, Germany) were then inserted into the shoes that the participants wore (Fig. 1). The IMUs (XsensDOT; Movella Inc., NV, USA) were attached to the dorsum of both shoes. The sampling frequencies were set to 100 Hz for the insole force sensors and 120 Hz for the IMUs. Because insole force sensors can measure three regions of the foot, the vertical GRFs at the hindfoot, midfoot, and forefoot of each foot were recorded. Foot acceleration and angular velocity were recorded using IMUs.

## Muscle strength

Maximal isometric KES was assessed using the Biodex System 4 (Biodex Medical Systems, Inc., NY, USA). Because it has been reported that lower isometric KES increase the risk of incident radiographic knee osteoarthritis in women^2^, we focused on isometric KES. The participants were seated with their hips flexed at 90° and tested at a knee flexion angle of 60°. The participants crossed their arms and placed the arms in front of their chests. Each participant performed maximal voluntary isometric knee extensor contraction. All participants were verbally instructed to push “as hard as possible” until the torque reached a plateau or decreased from the peak (approximately 5 s) and were provided a 30-second recovery period^12^ between trials. The researcher provided strong verbal encouragement during each trial. Three trials were performed on each limb. Prior to testing, participants completed several practice trials to familiarize themselves with the procedure. Maximal isometric KES was defined as the highest knee extensor torque recorded across three trials, and the highest values from both legs were averaged. The maximal isometric KES was normalized to body mass.

### Data analysis

#### Insole force sensor

The vertical GRFs of the three regions were summed to obtain the total vertical GRF acting on the feet. Gait events (heel contact and toe-off) were determined by using a vertical GRF threshold of 25 N^37^. Vertical GRFs were filtered using a second-order Butterworth low-pass filter with a zero-phase lag and cutoff frequency of 20 Hz^37,38^. Sixty gait cycles were analyzed for each participant (10 one-way walks [5 round trips] × 6 gait cycles/pathway [3 gait cycles per leg]). That is, the gait cycles of the left and right lower limbs were analyzed regardless of limb dominance.

**Fig. 2:**
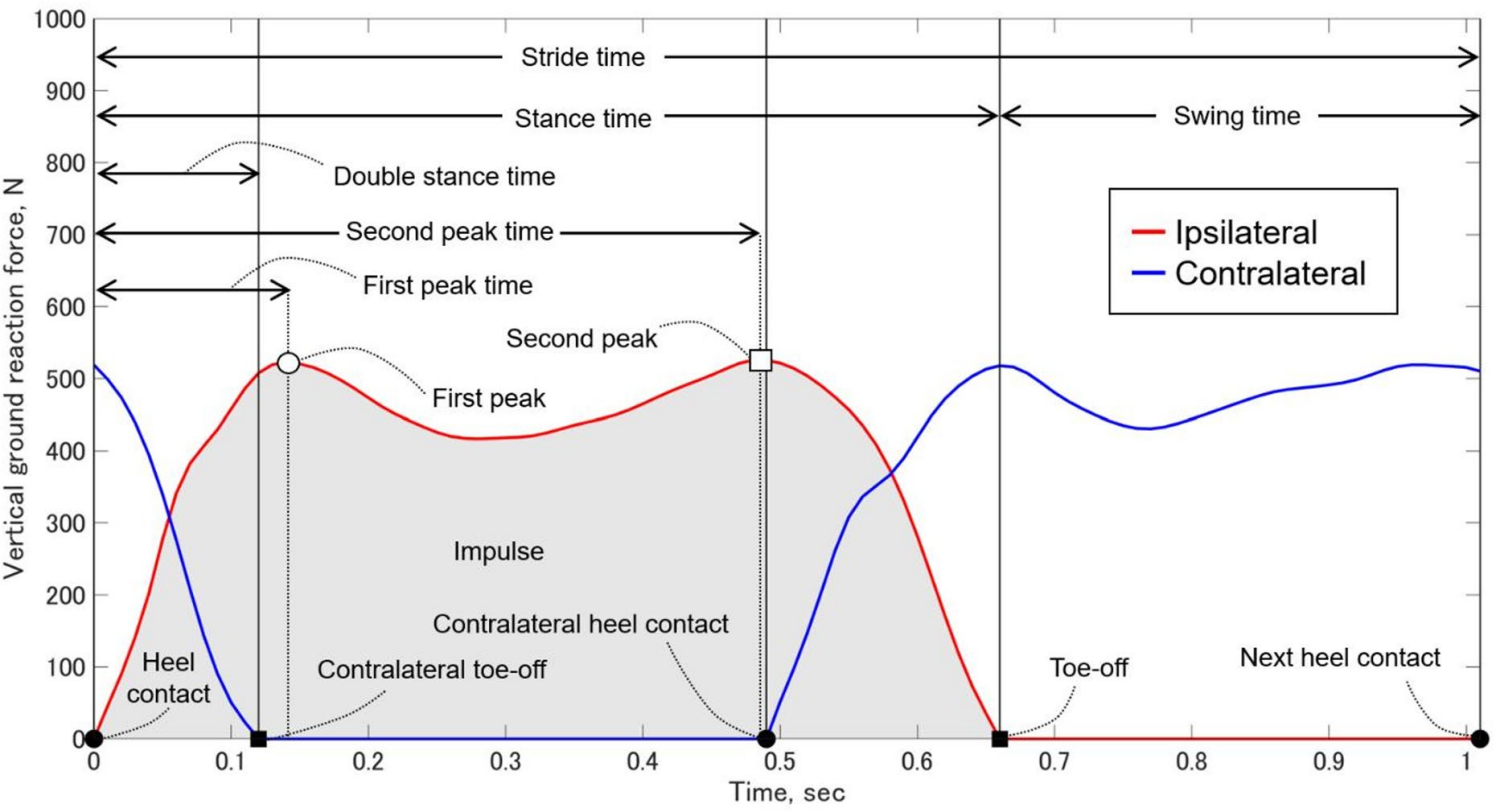
Temporal and force parameters calculated using vertical GRF data. The gray area indicates the impulse of the vertical GRF. The black circles and squares indicate the heel contact and toe-off, respectively.

Figure 2 illustrates definitions of the temporal and force-related parameters derived from the insole force sensor data. Cadence was calculated using stride time (60 × 2 / stride time). The stride time was defined as the time between heel contact and subsequent ipsilateral heel contact. Stance time was defined as the time from heel contact to ipsilateral toe-off, while swing time was defined as the time from toe-off to the subsequent ipsilateral heel contact. Double-stance time was defined as the time from heel contact to the subsequent contralateral toe-off (i.e., the first double-stance period in a gait cycle). The percentages of the stance, swing, and double-stance phases were defined as their respective times relative to the stride time. In addition, we focused on the first double-stance time rather than calculating the sum of the first and second double-stance times to clarify the temporal relationship between the end of the first double-stance phase and the timing of the first peak vertical GRF, and to provide information on the relationship for readers. The first and second peak vertical GRFs were calculated as the maximum peaks within 0–50% and 50–100% of the gait cycle, respectively, and both values were normalized to body mass. The first and second peak times were defined as the times at which the first and second peak vertical GRFs occurred, respectively, and their timings were expressed relative to the stride time. The vertical GRF impulse was calculated by integrating the vertical GRF over time during the stance phase, and the value was normalized to body mass.

## IMU sensor

**Fig. 3:**
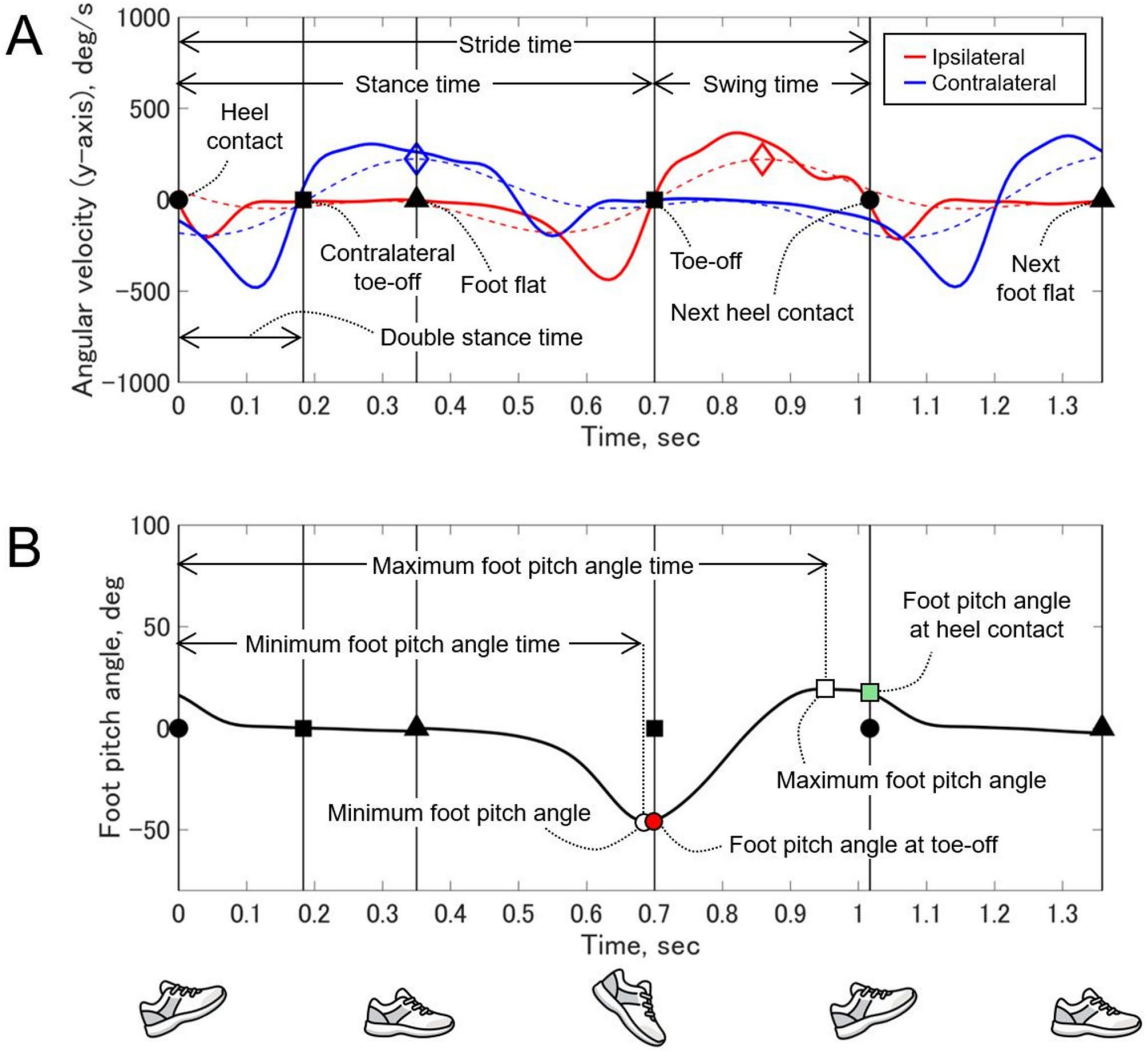
Temporal and foot pitch angle parameters calculated using IMU data. a: Zero-crossing points of angular velocities (y-axis) were used to detect gait events (heel contact and toe-off). The dashed lines represent the two waveforms B, and the diamonds indicate the local maxima. b Positive and negative foot pitch angles indicate counterclockwise and clockwise rotations, respectively. Black circles, triangles, and squares indicate heel contact, flat foot, and toe-off, respectively.

According to a previous systematic review^39^, the zero-crossing method is reliable for detecting gait events (heel contact and toe-off). Therefore, based on a previous study, we adopted this method to identify these events^9^. Specifically, we first prepared two waveforms: (1) the y-axis angular velocity filtered using a fourth-order Butterworth low-pass filter with a zero-phase lag and a cutoff frequency of 10 Hz (waveform A) and (2) the y-axis angular velocity filtered using a fourth-order Butterworth low-pass filter with a zero-phase lag and a cutoff frequency of 2 Hz (waveform B). Local maxima (threshold > 70°/s^9^) were identified using waveform B. For waveform A. The zero-crossing points preceding a given local maximum were detected as toe-off events, whereas the zero-crossing point following the local maximum was detected as a heel-contact event (Fig. 3a). Foot flat was defined as the midpoint between heel contact and toe-off^9^.

The IMU data (three-axis acceleration and angular velocity for each foot) were filtered using a fourth-order low-pass Butterworth filter with a zero-phase lag and a cutoff frequency of 10 Hz^9^. Based on the filtered signals, the IMU orientation is calculated using the Kalman filter algorithm. Figure 1 illustrates the local coordinate system of the IMU sensors. For each shoe, the x-axis was oriented posteriorly, y-axis was oriented to the right, and z-axis was oriented superiorly (Fig. 1). For the global coordinate system, the direction opposite the gravitational vector during the foot flat (i.e., the first frame for each gait cycle from the foot flat to the subsequent ipsilateral foot flat) was defined as the Z-axis. The X-axis of the global coordinate system was defined as the projection of the x-axis of the IMU sensor coordinate system onto the horizontal plane while the foot was flat. The Y-axis of the global coordinate system was then determined based on the X- and Z-axes. Acceleration without the gravitational component $\:{\boldsymbol{b}}_{\mathrm{G}}$ was calculated using the following equation:

1 $$\:\begin{array}{c}{\boldsymbol{a}}_{\mathrm{G}}={{}^{\mathrm{G}}\boldsymbol{R}}_{\mathrm{S}}{\boldsymbol{a}}_{\mathrm{S}}\end{array}$$

2 $$\:\begin{array}{c}{\boldsymbol{b}}_{\mathrm{G}}={\boldsymbol{a}}_{\mathrm{G}}-\left[\begin{array}{c}0\\\:0\\\:g\end{array}\right]\end{array}$$

where $\:{\boldsymbol{a}}_{\mathrm{G}}$ represents the acceleration in the global coordinate system, $\:{\boldsymbol{a}}_{\mathrm{S}}$ represents the acceleration in the sensor coordinate system, $\:{{}^{\mathrm{G}}\boldsymbol{R}}_{\mathrm{S}}$ represents the rotation matrix from the sensor coordinate system to the global coordinate system, and $\:g$ represents gravitational acceleration. Gravitational acceleration was determined from 2.5 s of standing data for each participant. The velocity was calculated by integrating the acceleration. To reduce errors due to drift, a zero-velocity update^40^ was applied to each gait cycle (i.e., from the foot flat to the subsequent ipsilateral foot flat). The trajectories were calculated by integrating the corrected velocities. Furthermore, we assumed that the IMU heights at the foot flat and subsequent ipsilateral foot flat were the same^9^. Based on this assumption, the drift along the Z axis was corrected using the same method^40^.

Sixty gait cycles were analyzed for each participant (10 one-way walks [5 round trips] × 6 gait cycles/pathway [3 gait cycles per leg]). That is, the gait cycles of the left and right lower limbs were analyzed regardless of limb dominance. A single experimenter started the gait measurement by tapping the start buttons of the insole force sensor and IMU sensor applications simultaneously. However, strict time synchronization was not performed using these signals. Data were collected continuously over five round trips without starting or stopping the measurement for each one-way walk.

The spatiotemporal and kinematic gait parameters calculated in this study were as follows: (1) cadence, (2) stride time, (3) stance time, (4) swing time, (5) double-stance time, (6) percentage of stance phase, (7) percentage of swing phase, (8) percentage of double-stance phase, (9) gait speed (i.e., stride length divided by the time from foot flat to the subsequent ipsilateral foot flat^9^, (10) stride length (i.e., the distance between positions of the IMU at foot flat and the subsequent ipsilateral foot flat^9^, (11) walk ratio, (12) foot speed during the swing phase, (13) foot pitch angle at toe-off, (14) foot pitch angle at heel contact, (15) minimum foot pitch angle, (16) maximum foot pitch angle, (17) minimum foot pitch angle time, (18) maximum foot pitch angle time, (19) timing of minimum foot pitch angle, and (20) timing of maximum foot pitch angle. In addition, an offset was applied such that the foot pitch angle was 0° on the flat foot of each participant. Clockwise rotation was defined as a negative angle when the foot was viewed from the right side and counterclockwise rotation was defined as a positive angle. Except for (5) double-stance time, (7) percentage of swing phase, and (8) percentage of double-stance phase, these parameters were calculated based on a previous study^9^ (Fig. 2). Double-stance time was defined as the duration from heel contact to the subsequent contralateral toe-off (Fig. 2). The percentages of the swing and (first) double-stance phases were calculated as swing and double-stance times divided by stride time.

### Statistical analysis

Correlation analyses were performed to examine the associations between KES (and gait speed) and spatiotemporal, GRF, and kinematic parameters. First, the normality of each variable was assessed using the Shapiro–Wilk test. Depending on the distribution, Pearson or Spearman correlation coefficients were used to evaluate the relationships between variables. Furthermore, gait speed^13,14,16,25,28,29^ and age^29,41,42^ may act as confounding factors in the relationship between the KES and other gait parameters. Therefore, partial correlation analyses controlling for gait speed derived from the IMU sensors and age were also performed. In addition, we confirmed that the correlation coefficient between gait speed derived from the IMU sensors and that calculated from a motion capture system was 0.99 (Supplementary Fig. 1); therefore, we judged that it was suitable to use gait speed derived from the IMU sensors as a confounding factor. Statistical significance was set at *p* < 0.05. Additionally, effect sizes (Cohen’s *r*) were calculated and interpreted as negligible (*r* < 0.1), small (0.1 ≤ *r* < 0.3), medium (0.3 ≤ *r* < 0.5), and large (0.5 ≤ *r*) based on a previous study^43^.

## Results

The maximum isometric KES was 1.23 ± 0.25 Nm/kg. Table 1 shows the relationships between KES and the parameters calculated using the insole force sensors. Significant positive correlations were observed between the percentage of the swing phase and first-peak vertical GRF. Significant negative correlations were observed between the stance time, double-stance time, percentage of stance and double-stance phases, first and second peak times, timing of the first and second peaks, and vertical GRF impulse. Even after controlling for gait speed and age as confounding factors, significant positive correlations were observed for the percentage of the swing phase and significant negative correlations were observed for double-stance time, percentages of stance and double-stance phases, first peak time, and timing of the first and second peaks.

**Table 1:** Correlations of knee extensor strength and gait speed with parameters derived from insole force sensors (*n* = 37).

	Mean	(SD)	Knee extensor strength				Knee extensor strength				Gait speed			
			*p*-value	*r*	Effect size	Interpretation	*p*-value	*r*_*p*_	Effect size	Interpretation	*p*-value	*r*	Effect size	Interpretation
Cadence, steps/min	123.3	(7.6)	0.1432	0.25	0.25	Small	0.7344	0.06	0.06	Negligible	**0.0004**	0.55	0.55	Large
Stride time, sec	0.98	(0.06)	0.1366	-0.25	0.25	Small	0.7443	-0.06	0.06	Negligible	**0.0003**	-0.56	0.56	Large
Stance time, sec	0.63	(0.04)	**0.0128**	-0.41	0.41	Medium	0.1709	-0.24	0.24	Small	**0.0000**	-0.66	0.66	Large
Swing time, sec	0.35	(0.02)	0.4430	0.13	0.13	Small	0.2028	0.22	0.22	Small	0.2273	-0.20	0.20	Small
Double-stance time, sec	0.13	(0.02)	**0.0003**	-0.56	0.56	Large	**0.0079**	-0.44	0.44	Medium	**0.0000**	-0.67	0.67	Large
Percentage of stance phase, %	64.5	(1.6)	**0.0002**	-0.57	0.57	Large	**0.0048**	-0.47	0.47	Medium	**0.0030**	-0.47	0.47	Medium
Percentage of swing phase, %	35.5	(1.6)	**0.0002**	0.57	0.57	Large	**0.0048**	0.47	0.47	Medium	**0.0030**	0.47	0.47	Medium
Percentage of double-stance phase, %	13.5	(1.5)	**0.0002**	-0.58	0.58	Large	**0.0042**	-0.47	0.47	Medium	**0.0011**	-0.52	0.52	Large
First peak of the vGRF, N/kg	11.2	(1.2)	**0.0056**	0.45	0.45	Medium	0.2405	0.20	0.20	Small	**0.0000**	0.85	0.85	Large
Second peak of the vGRF, N/kg	9.4	(0.7)	0.1332	-0.25	0.25	Small	0.1549	-0.25	0.25	Small	0.5281	-0.11	0.11	Small
First peak time of the vGRF, sec	0.15	(0.02)	**0.0003**	-0.57	0.57	Large	**0.0093**	-0.43	0.43	Medium	**0.0000**	-0.68	0.68	Large
Second peak time of the vGRF, sec	0.5	(0.0)	**0.0230**	-0.37	0.37	Medium	0.1262	-0.26	0.26	Small	**0.0015**	-0.50	0.50	Large
Timing of the first peak of the vGRF, %	15.5	(1.7)	**0.0003**	-0.57	0.57	Large	**0.0074**	-0.45	0.45	Medium	**0.0012**	-0.51	0.51	Large
Timing of the second peak of the vGRF, %	48.0	(1.4)	**0.0297**	-0.36	0.36	Medium	**0.0092**	-0.43	0.43	Medium	0.8548	0.03	0.03	Negligible
vGRF impulse, Ns/kg	4.6	(0.4)	**0.0218**	-0.38	0.38	Medium	0.1540	-0.25	0.25	Small	**0.0011**	-0.51	0.51	Large

*Note*: Bold values indicate *p* < 0.05. rp: Partial correlation coefficient. vGRF: Vertical ground reaction force.

Table 2 shows the relationship between KES and the parameters calculated using the IMU sensors attached to the shoes. Significant positive correlations were observed for the percentage of the swing phase, gait speed, stride length, foot speed during the swing phase, foot pitch angle at heel contact, and maximum foot pitch angle. Significant negative correlations were observed for the stance time, double-stance time, percentage of stance and double-stance phases, foot pitch angle at toe-off, minimum foot pitch angle, minimum foot pitch angle time, and timing of minimum foot pitch angle. Even after controlling for gait speed and age as confounding factors, significant negative correlations were observed for the foot pitch angle at toe-off and minimum foot pitch angle.

**Table 2:** Correlations of knee extensor strength and gait speed with parameters derived from shoe-mounted IMU sensors (*n* = 37).

	Mean	(SD)	Knee extensor strength				Knee extensor strength				Gait speed			
			*p*-value	*r*	Effect size	Interpretation	*p*-value	*r*_*p*_	Effect size	Interpretation	*p*-value	*r*	Effect size	Interpretation
Cadence, steps/min	123.2	(7.7)	0.1391	0.25	0.25	Small	0.7156	0.06	0.06	Negligible	**0.0004**	0.56	0.56	Large
Stride time, sec	0.98	(0.06)	0.1321	-0.25	0.25	Small	0.7259	-0.06	0.06	Negligible	**0.0002**	-0.57	0.57	Large
Stance time, sec	0.66	(0.05)	**0.0391**	-0.34	0.34	Medium	0.5095	-0.12	0.12	Small	**0.0000**	-0.70	0.70	Large
Swing time, sec	0.32	(0.02)	0.9509	-0.01	0.01	Negligible	0.8653	0.03	0.03	Negligible	0.3101	-0.17	0.17	Small
Double-stance time, sec	0.17	(0.02)	**0.0044**	-0.46	0.46	Medium	0.1610	-0.24	0.24	Small	**0.0000**	-0.83	0.83	Large
Percentage of stance phase, %	67.7	(1.1)	**0.0043**	-0.46	0.46	Medium	0.1804	-0.23	0.23	Small	**0.0000**	-0.72	0.72	Large
Percentage of swing phase, %	32.3	(1.1)	**0.0043**	0.46	0.46	Medium	0.1804	0.23	0.23	Small	**0.0000**	0.72	0.72	Large
Percentage of double-stance phase, %	17.7	(1.1)	**0.0036**	-0.47	0.47	Medium	0.1510	-0.25	0.25	Small	**0.0000**	-0.71	0.71	Large
Gait speed, m/s	1.12	(0.16)	**0.0096**	0.42	0.42	Medium	NA	NA	NA	NA	NA	NA	NA	NA
Stride length, m/HT	0.73	(0.08)	**0.0174**	0.39	0.39	Medium	0.9211	-0.02	0.02	Negligible	**0.0000**	0.91	0.91	Large
Walk ratio, mm/(steps/min)	4.5	(0.6)	0.1950	0.22	0.22	Small	0.6187	-0.09	0.09	Negligible	**0.0001**	0.59	0.59	Large
Foot speed during the swing phase, m/s	1.28	(0.19)	**0.0090**	0.42	0.42	Medium	0.7518	0.06	0.06	Negligible	**0.0000**	1.00	1.00	Large
Foot pitch angle at toe-off, deg	-53.4	(4.9)	**0.0003**	-0.56	0.56	Large	**0.0130**	-0.42	0.42	Medium	**0.0006**	-0.54	0.54	Large
Foot pitch angle at heel contact, deg	26.0	(5.3)	**0.0273**	0.36	0.36	Medium	0.7455	0.06	0.06	Negligible	**0.0000**	0.77	0.77	Large
Minimum foot pitch angle, deg	-55.6	(5.2)	**0.0004**	-0.56	0.56	Large	**0.0182**	-0.40	0.40	Medium	**0.0001**	-0.62	0.62	Large
Maximum foot pitch angle, deg	28.2	(5.3)	**0.0269**	0.36	0.36	Medium	0.7948	0.05	0.05	Negligible	**0.0000**	0.79	0.79	Large
Minimum foot pitch angle time, sec	0.64	(0.05)	**0.0423**	-0.34	0.34	Medium	0.5943	-0.09	0.09	Negligible	**0.0000**	-0.73	0.73	Large
Maximum foot pitch angle time, sec	0.95	(0.06)	0.1431	-0.25	0.25	Small	0.7316	-0.06	0.06	Negligible	**0.0003**	-0.56	0.56	Large
Timing of minimum foot pitch angle, %	65.5	(1.3)	**0.0072**	-0.43	0.43	Medium	0.3694	-0.16	0.16	Small	**0.0000**	-0.83	0.83	Large
Timing of maximum foot pitch angle, %	97.1	(0.5)	0.7883	-0.05	0.05	Negligible	0.8690	-0.03	0.03	Negligible	0.3814	-0.15	0.15	Small

*Note*: Bold values indicate *p* < 0.05. rp indicate partial correlation coefficient. HT: Height. NA: Not applicable.

## Discussion

We investigated the associations between KES and spatiotemporal, GRF, and foot pitch angle parameters derived from wearable sensors attached to shoes in older women. Our two hypotheses regarding stride length and first peak of the vertical ground reaction force were supported. However, focusing on the magnitude of the effect sizes, the main findings of this study are summarized as follows: (1) KES was associated with the percentage of the stance, swing, and double-stance phases calculated from both the insole force and IMU sensors (medium to large effect sizes); (2) KES was associated with the timing of the first peak of the vertical GRF measured by the insole sensors (large effect size); and (3) KES was associated with the foot pitch angle at toe-off obtained from the IMU sensors attached to shoes (large effect size), and (4) the percentages of the stance, swing, and double-stance phases and the timing of the first peak of the vertical GRF derived from the insole force sensors, as well as the foot pitch angle at toe-off (and the minimum foot pitch angle) derived from the IMU sensors, were significantly associated with KES independently of gait speed and age (medium effect sizes). Although previous studies have measured the KES and gait characteristics^12–26^, to our knowledge, no study has examined these associations (simple and partial correlation analyses) using wearable sensors (insole force sensors and shoe-mounted IMU sensors) during comfortable walking in older women. Therefore, our study is first to examine these associations and provides valuable insights that may contribute to the estimation and quantification of KES from natural daily walking. We consider that our findings are novel in that they (1) identify which parameters derived from wearable sensors exhibit larger effect sizes (i.e., stronger associations with KES) among various sensor-based parameters, and (2) clarify which parameters are associated with KES independently of gait speed.

Simple correlation analyses revealed that the KES was negatively associated with the percentages of stance and double-stance phases, as measured by both insole force sensors and IMU sensors. Previous studies have reported positive associations between the KES and gait speed^14,25^, and negative associations^44,45^ between gait speed and the percentages of the stance and double support phases. Similar associations were observed herein. We believe that these results can explain the negative correlations between the KES and the percentages of the stance and double-stance phases. Furthermore, partial correlation analyses revealed that the KES was associated with the percentages of the stance and double-stance phases derived from the insole sensors, independent of gait speed and age. One possible explanation is that the quadriceps contribute to balance control^46^; a reduced KES may be a compensatory strategy to enhance stability during walking. This observation is supported by previous studies reporting that (1) muscle weakness (KES) is associated with poorer balance ability^46^, (2) reduced balance affects longer double-stance times^47^, and (3) muscle weakness (including in the quadriceps) promotes conservative gait strategies^48^ (e.g., increased stability margin). As a result, the increase in the percentages of the stance and double-stance phases may have been caused by a reduction in gait speed due to decreased KES and by a decline in balance ability resulting from decreased KES.

We found that a weaker KES was associated with later timing of the first peak of the vertical GRF, and the association remained even if gait speed and age were used as confounding factors. This association may be partially explained by the negative correlation observed between the percentage of the double-stance phase and their KES scores. Typically, the first peak of the vertical GRF occurs immediately after toe-off of the contralateral limb^49^, reflecting the transition from a phase in which body weight is supported by both limbs to a phase in which it is supported by the ipsilateral limb alone. Herein, the percentage of the double-stance phase was 13.5%, and the timing of the first peak of the vertical GRF was 15.5% (Table 1). We consider that a weaker KES may have led to a longer double-stance phase, which contributed to the delayed timing of the first peak of the vertical GRF.

According to previous studies, KES is associated with gait speed^14,25^, and gait speed is related to the ankle plantar flexion angle at toe-off^29^. In the present study, we demonstrated a significant association between KES and gait speed, as well as between gait speed and foot pitch angle at toe-off (and the minimum foot pitch angle). Although causal relationships could not be established from our cross-sectional data, these findings suggest that a decline in KES may reduce gait speed, which in turn may alter the foot pitch angle at toe-off. Furthermore, because the foot pitch angle at toe-off (and the minimum foot pitch angle) remained associated with KES even after controlling for gait speed and age, our results suggest the existence of a pathway that is independent of gait speed. To clarify this mechanism, computer simulations that allow the examination of causal relationships, such as forward dynamic simulations^48^, may be useful. Specifically, by constructing two musculoskeletal models, one representing knee extensor muscle weakness and the other representing healthy muscle function, and simulating walking at the same gait speed in both models, the effect of knee extensor muscle weakness on foot pitch angle at toe-off while controlling for gait speed can be evaluated. This approach provides valuable insights into the direct effects of reduced KES on gait kinematics.

Even after controlling for gait speed and age as confounding factors, the KES was significantly associated with temporal gait parameters (double-stance time and percentages of the stance, swing, and double-stance phases) derived from insole force sensors, first peak time, timing of the first and second peaks, and kinematic parameters (foot pitch angle at toe-off and minimum foot pitch angle) calculated from the IMU sensors. Gait speed can be influenced by various factors (e.g., cognitive function^50^, fear of falling^51^, and pain^52)^, and changes in gait speed can alter gait parameters^28^ and kinematics^28,29^. In other words, even if the KES does not decline, the gait parameters and kinematics may change owing to other factors. However, our results demonstrated that these gait parameters and kinematics were associated with KES independently of gait speed. Therefore, even if gait speed is altered by factors other than KES, focusing on these gait parameters (e.g., percentage of the double-stance phase) and kinematic variables (e.g., foot pitch angle at toe-off) may allow for a more accurate estimation of KES, which is the contribution of this study.

Our goal is to monitor KES using daily walking data. Therefore, as an additional analysis, we examined how accurately KES can be estimated from the data obtained from the insole force sensors and IMU sensors (Supplementary Table 1). Correlation coefficients between the measured and estimated KES values were calculated. When the parameters derived from both the insole force and IMU sensors were used, the correlation coefficient was 0.53. When only the insole force sensors or only the IMU sensors were used, the correlation coefficients were 0.50 and 0.35, respectively. Although these correlation coefficients were not high, our additional analysis results (Supplementary Table 1) suggest that parameters derived from sensor data may be helpful for estimating the KES. According to a previous study^53^, the association between the KES and gait speed is stronger at fast walking speeds than at comfortable walking speeds. Herein, we focused only on comfortable walking speeds; however, incorporating walking at maximal speed may enable a more accurate estimation of KES. Therefore, examining the relationship between KES and gait parameters during maximal-speed walking may be important for future research.

In the present study, muscle strength asymmetry was not included as an exclusion criterion. To examine the potential influence of muscle strength asymmetry on our results, we evaluated KES asymmetry for each participant. Specifically, based on a previous study^54^, KES asymmetry was calculated (Supplementary methods), and participants with an asymmetry of ≥ 20% were classified as having asymmetric KES. Six of them were identified as having asymmetric KES. We then excluded these six participants and reanalyzed the correlations, partial correlations, and accuracy of the KES estimations (Supplementary Tables 2–4). The results were almost identical to those obtained from the analyses that included all 37 participants. Therefore, although six participants with asymmetric KES were included in the main analysis, we believe that their influence on the main findings was minimal.

This study has some limitations. First, the differences in walking environments should be considered. Herein, the participants performed walking trials in a controlled laboratory setting. Previous research^55^ indicates that walking speed differs between laboratory-based and daily walking; consequently, other parameters may vary depending on the environment. Future studies should collect daily walking data using wearable sensors attached to shoes and examine their association with the isometric KES. Second, the participants wore standardized shoes during the test. As footwear differences have been reported to influence gait parameters^56^, it is important to investigate the relationships between KES and spatiotemporal, GRF, and kinematic parameters while the participants wear their usual shoes. Finally, we did not perform radiographic examinations to confirm whether the participants had orthopedic conditions that could directly affect the KES (e.g., knee osteoarthritis). Therefore, older women with radiographic knee osteoarthritis could have been included in the sample. Because gait characteristics have been reported to differ between individuals with knee osteoarthritis and healthy older adults^57^, future studies should stratify participants according to the presence or absence of knee osteoarthritis and examine the associations between sensor-derived parameters and KES separately in each group.

## Conclusion

In this study, we examined the associations between maximal isometric KES and spatiotemporal, GRF, and kinematic parameters derived from wearable sensors placed in the shoes worn by elderly women. Our results demonstrated that the KES was significantly associated with temporal parameters, including the percentages of the stance and double-stance phases. In addition, a lower KES was related to delayed timing of the first peak of the vertical ground reaction force and a smaller foot pitch angle at toe-off. Importantly, the significant associations between the KES and temporal, GRF-related temporal parameters derived from the insole force sensors, as well as the kinematic parameters obtained from the IMU sensors, persisted after adjusting for gait speed and age. Collectively, these findings suggest that specific temporal and kinematic parameters obtained from shoe-mounted wearable sensors reflect variations in the maximal isometric KES during natural walking. Such sensor-derived parameters may therefore have potential utility for the noninvasive estimation of KES in older women.

## Electronic Supplementary Material

Below is the link to the electronic supplementary material.

Supplementary Material 1

## Data Availability

Our data are not publicly available to protect participants’ individual identification codes. These datasets are available from the corresponding author upon request.
