# Supplementary material for: Associations between knee extensor strength and gait characteristics derived from shoe-based wearable sensors in older women
Source: Sci Rep. 2026 May 3;16:20447. doi: 10.1038/s41598-026-51573-0 (PMC13332041; doi:10.1038/s41598-026-51573-0)
Supplement: Supplementary file 1 — Supplementary Material 1 [file 41598_2026_51573_MOESM1_ESM.docx]

**Supplementary materials**

**Validation of gait speed and stride length derived from shou-mounted IMU sensors**

**Methods**

We evaluated the association between gait speed and stride length derived from shoe-mounted IMU sensors and those obtained using a gold-standard motion-capture system. In the present study, reflective markers were placed at the midpoint between the left and right posterior superior iliac spines, left and right heels, and midpoints between the second and third metatarsal heads on both feet. The center of each foot was defined as the midpoint of the line connecting the heel marker and the midpoint between the second and third metatarsal head markers. The velocity at the center of the foot was then calculated. Based on a previous study^1^, heel contact events were identified using the vertical component of the velocity of the foot center. Each participant performed five rounds of walking trips. From the motion capture data, two gait cycles (left and right) were analyzed for each one-way walk. Consequently, 20 gait cycles were analyzed per participant, and gait speed and stride length were calculated for each gait cycle. Finally, correlation coefficients were calculated to confirm the associations between the gait speed and stride length obtained from the motion capture system and those derived from the IMU sensors.

**Results**

**
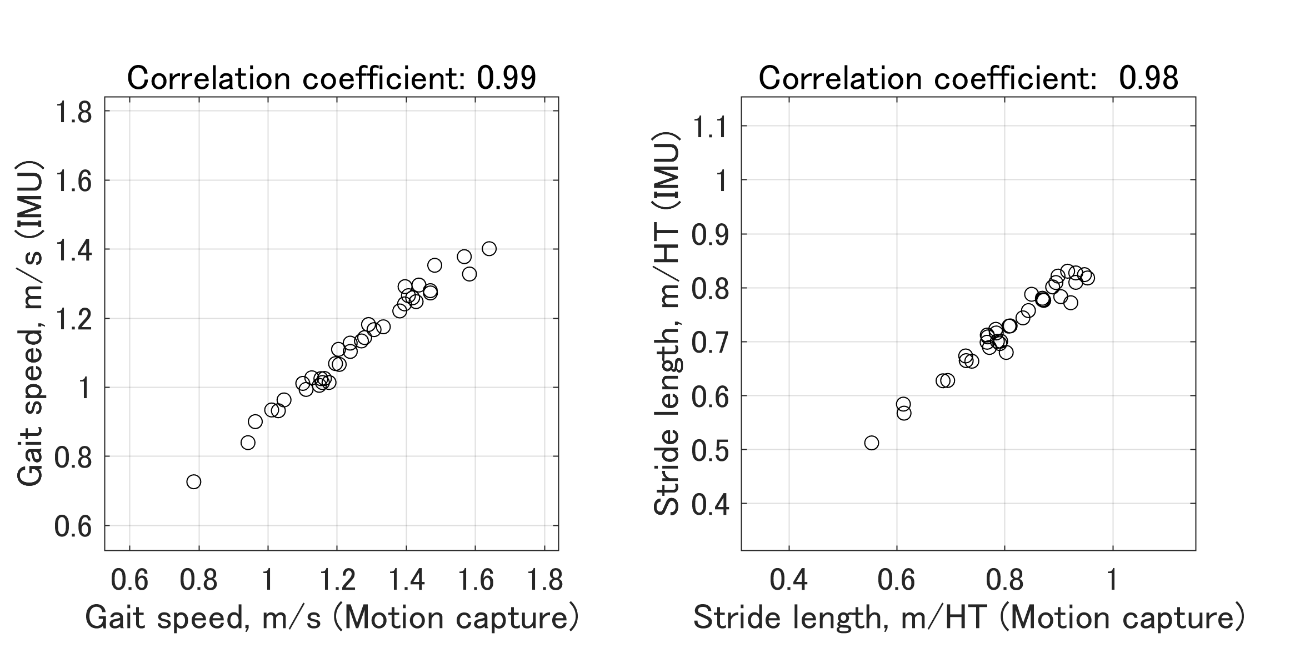
**

**Supplementary Figure 1.** Correlations for gait speed and stride length between IMU sensors and the motion capture system.

**Estimation of knee extensor strength**

**Methods**

The KES was estimated by using a combination of principal component analysis (PCA) and multiple linear regression analysis, based on parameters derived from insole force sensors and/or inertial measurement units (IMUs). A total of 37 participants were included in the analysis. A leave-one-out cross-validation procedure was used to evaluate the generalizability of the estimation model, a leave-one-out cross-validation (LOOCV) procedure was employed. In each iteration, data from one participant were excluded as test data, and the remaining data (training data) from the 36 participants were used to construct the estimation model. This procedure was repeated until each participant had served as a test case.

The parameters per condition (Condition: (1) insole force sensors and IMUs, (2) insole force sensors, and (3) IMUs) were used for PCA. Principal components with eigenvalues ≥ 1 were retained. To perform feature selection, Pearson’s correlation coefficients were calculated between each PCS and the measured KES within the training dataset. Only principal components whose absolute correlation coefficient with KES was ≥ 0.3, corresponding to at least a moderate effect size, were selected as explanatory variables for the regression model.

Multiple linear regression analysis was subsequently performed using the selected standardized PCSs as independent variables and the KES as the dependent variable. The resulting regression model was then applied to the excluded participants by projecting the test data onto the principal component space derived from the training data and calculating the corresponding PCSs using the same centering, scaling, and rotation parameters. These PCSs were entered into the regression model to obtain the estimated KES values for the test participants. After completing all the LOOCV iterations, the estimated KES values were obtained for all 37 participants. The model performance was evaluated by calculating the absolute error between the estimated and measured KES values, as well as the Pearson correlation coefficient between the estimated and measured KES values.

**Results**


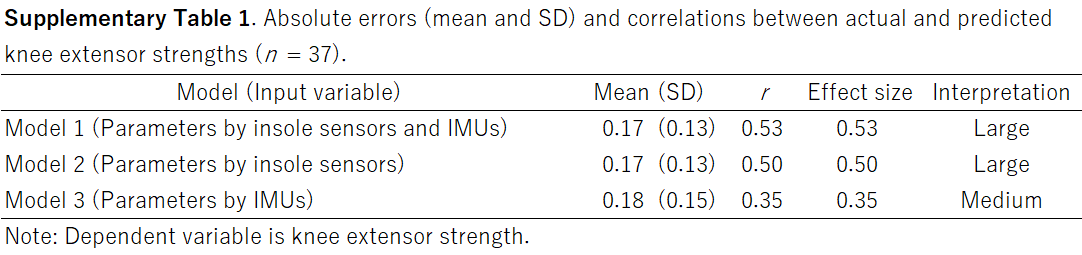


**Calculation of knee extensor strength (KES) asymmetry**

**Methods**

An asymmetry^2^ of KES was calculated for each participant using the following equation.

$$Asymmetry=\frac{\left| Weak leg value-Strong leg value \right|}{Strong leg value}\times100$$

The asymmetrical cutoff was set at 20% based on a previous study.^2^

**Results**

The asymmetry was 12.3 ± 8.7% (minimum and maximum values were 0.21% and 35.7%, respectively) in the present study. Of the 37 participants, six exhibited asymmetries greater than 20%.

**References**

1. O’Connor, C. M., Thorpe, S. K., O’Malley, M. J. & Vaughan, C. L. Automatic detection of gait events using kinematic data. *Gait Posture* **25**, 469–474 (2007).

2. LaRoche, D. P., Cook, S. B. & Mackala, K. Strength Asymmetry Increases Gait Asymmetry and Variability in Older Women. *Med Sci Sports Exerc* **44**, 2172–2181 (2012).
